# Academy of Medicine

**Published:** 1880-11

**Authors:** 


### ACADEMY OF MEDICINE

*Hœmorrhophilia.*—Dr. Whittaker read an essay on hæmorrhophilia published in full in a previous number of the *Lancet and Clinic.*

Dr. Forchheimer reported the history of a family of bleeders, or hæmophiles, occurring in his own practice.

The father was a bleeder, but died fourteen years after marriage from catarrhal pneumonia. The mother gave no evidence of hemorrhagic diathesis. This couple had nine children, four of which had been treated at various times by the speaker,the bleeders being all boys and no girls. The first child, when seven years of age, had a severe attack of epistaxis, following a blow received on the nose. Since that time this child had frequent and profuse attacks of hemorrhage, and died eventually of œdema of the lungs during an attack of scarlatina, during the course of which it frequently bled from the nose, and presented evidences of hemorrhagic effusion under the skin. Another boy in the same family had also frequent hemorrhages under the skin, but has enjoyed an immunity from hæmophilia for the last six months.

Dr. Cleveland mentioned an instance where epistaxis terminated fatally in an adult female, when prostrated by typhoid fever. A sister of this patient was likewise subject to frequent attacks of hemorrhage. Speaker saw another case where excessive hemorrhage followed a blow on the mouth. He would like to know if hæmophilia bore any relationship to pthisis, as the cases mentioned were both suffering from this disease.

Dr. Ransohoff mentioned a most interesting register of a family of bleeders in Germany. The grandfather was entirely well and had ten children—six boys and four girls. All the boys developed the hæmophile tendency, while the girls were free from it. In the second generation nineteen children were born to one family and thirteen to the other, until finally in 100 persons there were seventeen hæmophiles, and, singularly enough, not one in the fifth generation. Speaker considered its rarity due to the fact that the disease gradually dies out. Saw once a case of pupura urticans with the hemorrhagic diathesis in which there was no hereditary traceable. In answer to a question of the previous speaker would state that the history of a case reported in France, where all the members of a family of bleeders succumbed to pthisis after repeated hemorrhagic fluxes, would show a close relationship existing between the hemorrhagic diathesis and tuberculosis.

Dr. Wright asked whether in the cases recorded there was any strumous diathesis. He had such a case at present under treatment. The father had violent epistaxis and bleeding sores on the leg. The patient, a daughter, has had several attacks of uterine hemorrhage occurring as often as three or four times a day. She married at the age of twenty-one; but this diathesis continued even after she had become pregnant. One week before confinement, a small spot showed itself on a finger of the left hand. This speck, although only the size of a pin's head, ruptured and bled violently, and yielded finally only to continued compression. Another sister was similarly affected, but in both cases there was a strumous diathesis.

Dr. Reamy remarked that if hæmophilia be transmissable only be hereditary influence, he doubted if he ever had seen a true case of this disease, although he witnessed numerous instances of profuse hemorrhage following trivial causes, in which there was no evidence of heredity. A gentleman suffered in his childhood from repeated attacks of bleeding, but grew up in good health, until his eighteenth year, when he bled profusely from the nose. The nostrils were plugged, but hemorrhage occurred in different parts of the body, and the patient finally died. The father of this patient suffered occasionally but not excessively from attacks of epistaxis. Another case, a young lady, died from hemorrhage of the gums after repeated attacks of bleeding. Saw also several children succumb to hemorrhage of the nose, gums and around the toenails. Speaker thought if bleeders were so fruitful, an average of nine children being born to each family, and this hemorrhagic diathesis be transmitted more by females than males, it was strange that the majority marrying had no history of hereditary hemorrhage. Consequently this particular type of hemorrhagic diathesis has not been satisfactorily proven as being a hereditary affection. Speaker added that the extraction of teeth is followed frequently by obstinate hemorrhage. The smaller blood-vessels are the parts affected and not the larger vessels, consequently capital operations in hæmophiles are not followed by fatal hemorrhage any more frequently than is healthy individuals.

Dr. Ransohoff considered the immunity from hemorrhage in capital operations, where the larger blood vessels are divided, due to the fact that hæmophilia occurs in distinct intermission and the operations were performed during one of these.

Dr. Reamy replied that if this were the case the whole pathology of this disease would be set at naught, since, if these be defective, vascular structures as an inheritance of organization, the defect must continue between the hemorrhagic attacks.

Dr. Whittaker closed the discussion, remarking that every statement of the pathology of the disease yet advanced was inadequate to explain all the cases. The vascular theory which he heard advocated in his paper could not cover every case, nor would it explain the periods of immunity from hemorrhage, which "bleeders" enjoy. But one point the speaker would insist upon as prerequisite to the contraction of this strange condition, and that was the separation from it of all conditions which stimulate it. Bleeding from trivial cause were in many conditions, as in purpura, scurvy, leucocythæmia, pernicious anæmia, etc.; but the bleeding in these cases is the only symptom in connection with hæmophilia. For, in all these cases the condition is acquired; in some it is a result of a known cause of degradation, as in scurvy, anæmia and leucocythæmia, but in hæmophilia there is no cause, and hence no question of acquisition. Hæmophilia is a question of heredity, and this is the case in allied affections. The speaker did not venture to explain the most frequent escape of the attack on the part of the female. During sexual life, it might be said that the menses offered outlet for superflous blood; but the exemption was noticed before menstruation was established. Questions of heredity we do not yet attempt to decide. We cannot say why a man has a beard and a woman has not, or why is the difference between the sexes. We satisfy ourselves as yet with heredity as with a convenient shelter. Hæmophilia is, therefore, not, strictly speaking, hemorrhagic diathesis, because one may and does often acquire such a diathesis as the result of severe acute disease, typhus, icterus, gravis, etc.

Hæmophilia is a condition apart from all these states because it is inherited, and a case of hemorrhage which is not inherited is not hæmophilia.—*Extract from Cincinnati Lancet and Clinic.*

